# Supplementary material for: Loop-extruders alter bacterial chromosome topology to direct entropic forces for segregation
Source: Nat Commun. 2024 May 30;15:4618. doi: 10.1038/s41467-024-49039-w (PMC11139863; doi:10.1038/s41467-024-49039-w)
Supplement: Supplementary file 3 — Description of Additional Supplementary Files [file 41467_2024_49039_MOESM3_ESM.pdf]

Description of Additional Supplementary Files

File Name: Supplementary Movie 1
Description: Animations of replication simulations without loop-extruders and no *ori*-pulling. The blue and orange correspond to newly replicated strands, whereas the black line corresponds to the unreplicated part of the chromosome.

File Name: Supplementary Movie 2
Description: Animations of replication simulations with loop extruders and no *ori*-pulling. The blue and orange correspond to newly replicated strands, whereas the black line corresponds to the unreplicated part of the chromosome.

File Name: Supplementary Movie 3
Description: Animations of replication simulations without loop-extruders and with *ori*-pulling. The blue and orange correspond to newly replicated strands, whereas the black line corresponds to the unreplicated part of the chromosome.

File Name: Supplementary Movie 4
Description: Animations of replication simulations with loop-extruders and *ori*-pulling. The blue and orange correspond to newly replicated strands, whereas the black line corresponds to the unreplicated part of the chromosome.
